# Italian Children’s Accounts of the Lockdown: Insights and Perspectives

**DOI:** 10.1007/s10826-022-02508-6

**Published:** 2023-01-10

**Authors:** Michele Capurso, Tiziana Pedale, Valerio Santangelo, Luciana Pagano Salmi, Claudia Mazzeschi

**Affiliations:** 1 Department of Philosophy, Social & Human Sciences and Education, University of Perugia, Perugia, Italy; 2 Neuroimaging Laboratory, IRCCS Santa Lucia Foundation, Perugia, Italy; 3 Department of Physiology and Pharmacology “Vittorio Erspamer”, Sapienza University of Rome, Rome, Italy; 4 Independent researcher, Perugia, Italy

**Keywords:** Covid-19, Lockdown experiences, Children, School reentry, Program, Narrative

## Abstract

COVID-19 lockdown-imposed restrictions emerged as a risk to children’s well-being. However, the extant literature often ignored children’s experiences, emotions, struggles, hopes, and expectations. Based on a large sample of Italian students (*N* = 906; mean age = 9.4 years, 48.8% female), we drew data from a post-lockdown school re-entry program where students completed narrative activities in 2020. These narratives underwent quantitative content analysis according to gender and school level. Overall, children reported mixed feelings about the lockdown; they felt safe at home but also experienced fear and missed their friends, school, and freedom. Screen-time, technology and friendships helped, but children struggled to make sense of the events. Our findings show how children attempted to make sense of the lockdown experience and may provide key information for the development of community coping programs to help children facing crises in the future.

## Highlights

- Children narrated their lockdown experiences as part of a post lockdown school re-entry program.
- We identified, coded, classified, and evaluated their narratives to examine perception and coping mechanisms utilized during the covid pandemic.
- Family, friendship, and relationships helped children to deal with the impacts of the pandemic and with the forced isolation.
- Children suffered from the loss of physical contacts with peers, freedom, and school.
- Understanding children’s experience during a crisis can benefit the future development of crisis coping programs.

The first wave of the COVID-19 pandemic hit the world between March and May 2020, impacting the lives of millions of children and young people. Approximately 94% of the world’s school population was affected, giving rise to the most significant disruption of education systems experienced in living history (UN Secretary-General, 2020). On March 4th, 2020, Italian children of all ages stopped going to school (Presidenza del Consiglio dei Ministri, 2020) and did not see their teachers and classmates personally until September 2020. The lockdown involved many social constraints including mobility restrictions, social distancing, self-isolation along with the prolonged closure of educational institutions, nonessential businesses, and a nationwide curfew (Haug et al., 2020). During this time, Italian youngsters had to cope with a large array of psychological stressors such as loneliness, insecurity, and instability in daily routines. Besides being isolated from peers and teachers, outdoor activities were very limited or banned (Cellini et al., 2021; Orgilés et al., 2020). Studies carried out during the lockdown show that this event affected the psychological state of approximately 79.4% of children who revealed symptoms of increased anxiety, depression, irritability, and inattention (Panda et al., 2020). Additionally, about one out of four children developed a significant fear of the virus, and about one-third of all children suffered from sleep disturbance (Panda et al., 2020). Such effects were mediated by a complex network of factors including culture (e.g., the way people think about, live, and cope with negative and positive emotions; Furlong & Finnie, 2020), socioeconomic status, health (Save the Children Italia, 2020), age, and gender (Davico et al., 2021). Feelings and emotions of fear, isolation, and boredom permeated children’s lives and their experiences of the pandemic transnationally. Some coping factors emerged as the result of cultural, social-cultural, and geographical peculiarities (especially in terms of lifestyle, education, participation in family life, communication, access and use of personal and open spaces, digital gap; Cortés-Morales et al., 2021).

Fortunately, children around the world also responded to the lockdown in more resilient ways. For example, one study showed that children’s positive emotions acted as mediators between their own and maternal coping strategies (Petrocchi et al., 2020). Testimonies reported by Stoecklin et al. (2021) suggested that, during the lockdown, children took the opportunity to spend their free time as they wished: spending more time with their families, starting new hobbies, taking care of themselves, and adopting a slower pace of life. In some cases, the pandemic outbreak stimulated children and young people to uphold their duties towards their family, community, and neighborhood (Jamieson & van Blerk, 2021). On a more developmental level, responses to stressors are known to change throughout life. They have been linked to the type of problem faced, to the individual’s appraisal of the situation, developmental stage, and gender (for a review, see Skinner & Zimmer-Gembeck, 2006). For example, a study by Eschenbeck et al. (2007) showed girls’ coping mechanisms more oriented towards seeking social support and problem-solving, whereas boys used more avoidant forms of coping. Crystal et al. (2008) outlined how children rely more on social support with increasing age and start shifting from parent-centered help to peer support, especially for emotional problems.

Everything considered, these studies show how children’s capabilities to face a crisis are connected to the dynamic interlink of contextual and individual variables. In this paper, we investigated how the first national lockdown of March 2020 affected Italian children’s experiences, emotions, struggles, hopes, and expectations as narrated by the direct voice of the protagonists themselves. This was possible through the structured narrative school-based activity. Our methodology differs from previous proxy-based studies of children, often reporting indirect observations on negative mental health outcomes of the pandemic in younger people (Samji et al., 2022). We employed an approach that corresponds with the principles of the United Nations Convention on the Rights of the Child (United Nations, 1989), which advocates for the active participation of children in events that affect them. Children participated as active individuals and agents in planning, preparedness, responses, and recovery efforts (Mutch & Marlowe, 2013). In addition, the present study was carried out within the children’s natural school environment.

## The Multiple Functions of Narratives and their Connections to Emotions

There are several recognized advantages to use narratives, especially with children (Kerry-Moran & Aerila, 2019). In this work, we used this approach for two main reasons.

In the first place, narratives reflect mental representations of an experience, together with the role that the self and others played in the event (Emde, 2003). In particular, anthropologist Edward Bruner (1984) differentiates between three categories of reality connected to the events and their narratives. The first one is “life as lived” (flow of events that touch a person’s life), the second is “life as experienced” (images, feelings, desires, and thoughts perceived by the person that are ascribed with a specific meaning), and the third one is “life as told” which, as a narrative, is inevitably influenced by the cultural conventions of telling, evidence, and the social context. Therefore, the narrative-based connection between what has happened and what is told is an active process. Through narratives, children organize the very essence of their life experience and find fundamental intra- and inter-personal ground in which they make sense of themselves, of the world around them, and of the relationship between the two (Engel, 2005). This process is not only centered on the past, it brings important consequences for the view of the future. As outlined by psychologist Jerome Bruner (1986), through storytelling, children can imagine how their existence might be or may anticipate future experiences and changes.

The other important reason for using narratives is that they play regulatory functions at a cognitive, social, and emotional level. When telling their experience, children integrate affect, cognition, and action related to the event (Favez, 2003), which in turn can change the very perception of the stated experience (Stern, 1985). Autobiographical narration within a social and interactive context contributes to constituting the self and promotes the acquisition of social meanings of the event (Favez, 2003). It is also connected to the child’s agency since telling allows the mind to see possible worlds and the different courses of action (Bruner, 1986). Especially in times of crisis, the chance to share one’s own emotions is a crucial process for functional regulation (Sala et al., 2014). Emde (2003) refers to this process with the term “emotional meaning-making” (p. 11). The emotion regulation hypothesis asserts that, when telling an event, children actively regulate their narrative with a double objective: to avoid facts that elicit negative emotions (Clyman, 2003) and to reappraise positive meanings and events, and these in turn modulate responses at an experiential, behavioral, or physiological level (Gross & John, 2003). In fact, according to Gross and John (2003), reappraisers process stressful events through an optimistic lens, reevaluating what they find stressful in an active effort to mend bad moods. As a result, they are more prone to sharing their emotions and have closer relationships with friends.

In the present paper, the two functions of narratives and the related meaning making processes became operational at two different times. The first one is for school re-entry intervention, which, in turn, generated the qualitative data that are then analyzed in the present research.

## A School Re-entry Intervention Program

Following the lockdown, a crucial aspect of the return to normality was represented by school re-entry (Fitzpatrick et al., 2020). If schools want to play a key role in students’ wellbeing, they need to recreate a classroom connection and a safe socio-emotional environment before engaging students in curricular work. Accordingly, we created a school re-entry program that used the student’s narratives with the double aim of sharing the different lockdown experiences and empowering children at a cognitive, social, and emotional level by processing, assessing, and discussing diverse events and strategies with their peers and teachers in the safe environment of the class. The program was built on 5 established crisis management principles for schools (Koplewicz & Cloitre, 2006; Theodore, 2016): to facilitate classroom discussions about the event, be open to feelings and uncertainty, provide opportunities for children to reconnect socially and with the environment, shift attention from the stressful memory to coping awareness, present facts and provide information to manage the situation.

The program included an online training module for teachers recognized by the Italian Ministry of Education, a manual for the teachers, and a set of activity packs for kindergarten, primary and middle school children. An English version of the program’s outline and related activity packs were made available via an open access article (Capurso et al., 2020) and are summarized here as part of the Supplementary File 1 & 2. The children’s booklet contained narrative and graphic activities focused on sharing and understanding emotions, fears, concerns, coping strategies, friendship empowerment, and icebreakers. The booklet ended with a space for the children to share a wish for how they would like to move forward at school.

According to the publisher’s metrics, since April 2020, the Italian and English version of the program together have been viewed or downloaded more than 90.000 times. The full program design, methods, measures, and results are described elsewhere (Capurso et al., 2021). As part of the program effectiveness evaluation, several classes tested the activities under the supervision of one of the authors (M.C.). Each student completed the booklet producing a set of personal narratives organized as a continuous storyline, starting at the lockdown period and ending in class when school restarted. This resulted in a unique sample of children’s accounts of their lockdown experiences.

The booklet was created along two axes. The first one is chronological: it starts from the past (“what happened during your lockdown”), acknowledges the present (“your re-found school life”), and it moves to wishful thinking for the future (“what will you do now that you are back to school”). The second is based on a developmental-contextualist view of human development. It traces different actors and processes in the child’s life (Ford & Lerner, 1992): family, school rooms, places, schoolmates, teachers, cognitions, emotions, social bonding, stressors, and coping responses.

## Narrative Self-reports of Children’s Experience

When children completed their booklet at school, they created a self-narrative of their experience. Narrative elicited self-reports are a key component of qualitative investigation with children (Engel, 2005). They involve children writing or recording their views, feelings, and experiences without direct and ongoing interaction with an interviewer. Such reports may include asking children to respond to scenarios and vignettes, questionnaires, sentence completion tasks, recording children’s naturally occurring narratives, and simple verbal prompts (Greene & Hogan, 2005). Self-reports fall under the general umbrella of *narratives*, i.e., representations of one or more events organized in an order which is assumed to show the sharer’s perspective of the event (Tsai, 2007).

## The Present Work

In terms of the COVID-19 lockdown, children were often analyzed from their parents’ perspectives (for a recent review, see Panda et al., 2020) and seen as passive subjects during the pandemic, excluded from actively contributing to the crisis management and response (Mutch & Marlowe, 2013). Contrary to that approach, the present study is based on the direct voices of children, who were actively working to understand, process, and share their own lockdown-related experiences, emotions, struggles, and hopes in their school context. Based on the collected narratives of primary and middle school students in Italy, and considering that literature reports differences in the way children face a crisis around the world (depending on age, gender, and culture), we addressed the following questions: what children reported to have missed the most and what helped them during lockdown (research question, RQ, 1), how the pandemic affected children’s spontaneous daily thoughts, concerns, and fears (RQ2), and what type of activities children wanted to do with their friends when they returned to school (RQ3). Additionally, we assessed whether there were differences based on gender or school level for all the RQs.

## Methods

### Study Design and Context

This study utilized a narrative-based, retrospective descriptive design to identify, observe, and measure different variables connected to the COVID-19 lockdown experience of a population of Italian children as soon as they re-entered schools. Data were extracted from the materials produced by the students during the school re-entry intervention program (i.e., the booklet from Capurso et al., 2020). The original intervention program aimed to empower children and teachers after the initial lockdown phase of the COVID-19 pandemic.

### Participants

In September 2020, when the Italian schools reopened for the first time after the COVID-19 lockdown, teachers and students from three school districts in the Umbria region of central Italy took part in our school re-entry program. After obtaining ethical approval, parental consent, and child assent, sixty classes (72% primary school, 28% middle school) took part in the program (Capurso et al., 2021) under the supervision of one of the authors. The program involved 54 teachers who administered it to 906 students (48.8% female, mean age 9.4 years, standard deviation (SD) 1.7 years, age range 7–13 years).

### Materials and Procedures

It should be noted that data were collected through teacher-guided school activities, and the researcher could not be present due to COVID protocols. After undergoing initial training, each teacher administered the activities to the pupils of their own classroom. Teachers were instructed to run the program as soon as possible after school reopened and to conclude it within a few days (the average duration was 5.1 days). Students were invited to complete a 7-page school re-entry booklet; they were free to skip any activity that they did not feel like doing (see Supplementary File 2 for the program’s outline and Capurso et al., 2020 for the children’s booklet). The booklets were collected and digitized before narrative data were extracted from excerpts for analysis. See Table 1 for a detailed list of the activity used by the children to answer the research questions of this study.

**Table 1:** Description of the school re-entry booklet activities that were retrieved and analyzed in the present research

Activity No. and name	Investigated Theme	Description	No. valid records
1. What did you miss?	Things most missed during the lockdown.	Direct questions: What did you miss most during the lockdown?	881
2. What helped?	Things that helped most during the lockdown.	Direct questions: What helped you most during the lockdown?	869
3. John and Mary’s thoughts	Concerns, worries, spontaneous thoughts about the coronavirus.	Students were invited to fill in a graphic vignette showing two children who “have heard of coronavirus from their parents and on television and have different thoughts”.	880
4. Back to school again!	Wishes for future activities at school and with friends.	Graphic vignette: children were invited to write what they would like to do with their school friends in the following few days.	846

See Supplementary File 2 for a detailed explanation of each activity, and Capurso et al., 2020 for the activity sheets

### Content Analysis

We used quantitative content analysis to analyze the children’s narratives (Huxley, 2020). This technique allows researchers to qualitatively and inductively determine constructs of interest and then to quantitatively assess their prominence concerning specific research questions (Krippendorff, 2004). This method was chosen because of the many short-sentence narratives generated in response to the booklet activities.

#### Development of a coding scheme

The classification of the responses followed an iterative process based on established contents analysis recommendations (Huxley, 2020; Krippendorff, 2004). The first and last authors qualitatively classified the analytical constructs by inductively assessing the participants’ open‐ended responses to develop the coding scheme. Then, they generated separate codebooks for each of the activities reported in Table 1. Different coding sections were created for activities with different prompts (e.g., what was missed most and what helped most; see Supplementary File 3 for the full codebook with examples). Subsequently, transcripts from 40 randomly selected participants were open‐coded by the first author and two research team members in order to generate preliminary codes. We collectively identified any word, phrase, or sentence believed to carry the same meaning, and these were grouped under the same label. These original codes consisted of a short label, a description, a definition of the concept, a list of criteria for inclusion or exclusion, and direct quotes from the participants for data excerpts. In this phase, atypical or uncommon answers (< 5%) were either grouped with other similar codes or were added to the “other” category. Once the initial codebooks had been generated, the answers were coded independently, and the authors met regularly to refine the definitions and codes. Summaries of the final codes, definitions, and exemplary quotes are presented in the attached codebook as Supplementary File 3. The original dataset (in Italian and Filemaker format) is available upon request from the corresponding author.

#### Coding of the transcripts

Using the finalized codebooks, two research team members independently coded all the children’s workbooks sections presented in Table 1. For each section, the analysis units were represented by the statements produced by children in response to the different prompts. These units were coded for the presence or absence of each code within each theme with the help of a Filemaker database that represented the coding sheet. If the same code appeared multiple times in the same response, it was only coded once. Interrater reliability analysis was performed by calculating the Cohen’s kappa (κ) for each code on the whole sample to corroborate the reliability of the coding. Results showed consistency values between 0.7 and 1 across codes. As a final stage, to maximize the data included in the subsequent analyses, we reviewed and merged the codes with comparable meanings or similar conceptual denotations. Upon completion of coding, code frequencies were tabulated.

### Coded Data Analysis

We aimed to understand children’s experiences of the lockdown and investigate any age or gender differences in the reported experiences. First, we tabulated the codes in order of prevalence to enable descriptive analyses of the children’s experiences. Codes that received fewer than 10% of responses in the entire group were excluded from further analyses. Second, to investigate whether the frequency of each code varied as a function of gender and school level, we performed a separate logistic regression for each code that had a prevalence in the entire group higher than 10%. For each logistic regression, the dependent variable was the dichotomous presence/absence of the code for each participant while the predictors were: the school level (between-subject factor with two levels; primary and secondary school age), gender (between-subject factor with two levels; male and female), and the school-by-gender interaction term. Statistical analyses were performed in R Studio for R version 4.0.2 (R Core Team, 2013). Logistic regressions were fitted using the generalized linear model (glm) function, assuming a binomial distribution, from the stats package (Dobson, 2006). Overall, the main effects of gender and school level and their interactions were checked using the likelihood-ratio chi-square test with the analysis of variance (ANOVA) function from the car package (Fox & Weisberg, 2018).

## Results

Example quotes to illustrate the key concepts narrated by children can be found for each code in the codebook included as part of the Supplementary File 3.

RQ1 assessed what children missed most (Table 2) and what helped them (Table 3) during the lockdown. To answer this RQ, activity 1 posed two direct open-ended questions. A total of 881 children reported what they missed most, and the data were included in our results (see Table 2). What was missed most during the lockdown in our sample were friendships and peers (88.08% of responders), followed by the school (70.17%), freedom, and autonomy, which included items such as “go out to play” and “hang out with friends” (37.46%), family and relatives (31.67%), sports and hobbies like “riding your bike” and “playing soccer” (22.70%), and “hanging out”, which, especially in Italian culture, is connected to hugging and physical and direct contact with others (15.55%).

**Table 2:** Students’ answers to the question “What did you miss most during the lockdown?” by school level and gender

Code	Full Sample % (*N* = 881)	Primary School		Middle School		Main effect of school		Main effect of gender		Interaction effect	
		Male % (*N* = 271)	Female % (*N* = 297)	Male % (*N* = 156)	Female % (*N* = 157)	χ^2^	*p*	χ^2^	*p*	χ^2^	*p*
Friendship and peers	88.08	83.39	85.19	94.23	95.54	24.29	**<0.001**	0.56	0.453	0.06	0.810
School	70.17	71.22	81.82	47.44	68.79	32.43	**<0.001**	22.83	**<0.001**	0.91	0.341
Freedom, autonomy	37.46	31.73	32.32	45.51	49.04	19.91	**<0.001**	0.26	0.612	0.16	0.692
Family, relatives	31.67	23.99	33.33	33.94	39.49	6.01	**0.014**	6.55	**0.011**	0.55	0.460
Sports, hobbies, physical activity	22.70	22.51	15.82	42.95	15.92	24.84	**<0.001**	11.73	**<0.001**	7.59	0.006
Hanging out, hugging, physical contact	15.55	8.12	14.81	20.51	24.84	18.66	**<0.001**	5.91	**0.015**	1.24	0.265
Play	8.63	12.92	7.07	6.41	6.37	nc	nc	nc	nc	nc	nc
Other (including pets, positive feelings)	5.68	7.01	5.39	5.77	3.82	nc	nc	nc	nc	nc	nc

Bold text shows *p*-values < 0.05

**Table 3:** Students’ answers to the question “What helped you most during the lockdown?” by school level and gender

Code	Full Sample % (*N* = 869)	Primary School		Middle School		Main effect of school		Main effect of gender		Interaction effect	
		Male % (*N* = 263)	Female % (*N* = 295)	Male % (*N* = 154)	Female % (*N* = 157)	χ^2^	*p*	χ^2^	*p*	χ^2^	*p*
Support from family members and other relatives	60.64	60.08	62.03	48.70	70.70	0.10	0.753	7.60	**0.006**	8.42	**0.004**
Video games, TV (passive use of ICT)	30.38	37.64	17.29	53.25	20.38	8.47	**0.004**	64.90	**<0.001**	1.79	0.181
School, distance learning	30.26	26.62	33.22	29.87	31.21	0.03	0.865	2.29	0.130	0.67	0.413
Active use of ICT	30.15	17.87	21.36	43.51	54.14	80.06	**<0.001**	4.16	**0.041**	0.43	0.512
Play	28.19	32.32	37.97	14.94	15.92	41.20	**<0.001**	1.78	0.183	0.23	0.633
Pets	17.26	14.83	20.00	17.53	15.92	0.08	0.771	1.14	0.285	1.59	0.207
Friendship	16.57	11.03	12.20	20.13	30.57	26.80	**<0.001**	3.29	0.070	1.39	0.237
Sports, hobbies, physical activity	14.38	15.59	12.20	16.23	14.65	0.40	0.529	1.32	0.250	0.17	0.683
Feeling useful, places and resources within the house	16.92	18.25	17.63	18.18	12.10	1.19	0.276	1.03	0.311	1.26	0.262
Other	11.39	12.17	9.15	12.99	12.74	0.98	0.323	0.88	0.348	0.46	0.498
Reading, music	7.48	2.28	8.81	9.74	11.46	nc	nc	nc	nc	nc	nc
Inner strength or self- identified resources	5.87	4.56	6.10	5.84	7.64	nc	nc	nc	nc	nc	nc

*ICT* Information communication technologies. Bold text shows *p*-values < 0.05

Moreover, there were significant differences in the occurrences of codes between primary (PSS) and middle school students (MSS) and between male and female children (see Table 2). All the codes revealed a main effect of school level; most of the reported activities were missed more in MSS compared to PSS (i.e., friendships and peers, freedom, family and relatives, sports and hobbies, and hanging out, and direct contact with others). Conversely, the unique code that was missed more in PSS compared to MSS was the school. There were also significant differences between gender; females missed school, family and relatives, hanging out, and having direct contact with others more than males. Males missed sport and hobbies the most. However, a significant interaction was detected in this last code; the difference between genders regarding the missing of sport and hobbies was more evident in MSS (males *vs*. females, 42.95% *vs*. 15.92%) than in PSS (males *vs*. females, 22.51% *vs*. 15.82%).

A total of 869 children responded to the question concerning what helped them most and were included in the following results (see Table 3). The children’s responses revealed the significant importance of the support from family members and other relatives (60.64%), followed by video games and television (30.38%), school and distance learning (30.26%), active use of information and communication technology, such as “chatting with friends” and “making a video” (30.15%), play activities (28.19%), pets (17.26%), friendships (16.57%), sport and hobbies (14.38%), and feeling useful in the house or the help from using specific resources from home (16.92%).

For this question, there were significant differences in the occurrence of the codes between PSS and MSS and between males and females (see Table 3). The code relating to support from the family was reported more by females than males, and there was a significant interaction between gender and school level, indicating that the difference between genders related to the importance of family support during the lockdown was more evident in MSS (females *vs*. males, 70.70% *vs*. 48.70%) than in PSS (females *vs*. males, 62.03% *vs*. 60.08%). Video games and TV helped males more than females, and MSS children than PSS children. In contrast, the active use of technology was of greater help for females than males, but again more for MSS than PSS. Finally, play was more important for PSS than MSS, while friendship was more helpful for MSS than PSS. School, pets, sport and hobbies, and domestic routines were equally reported across all school levels and genders.

RQ2 focused on children’s thoughts about the lockdown. RQ2 was based on an elicited narrative containing a vignette prompt, hence its “projective” nature (Chandler et al., 2003). Students were presented with a picture of two children surrounded by a set of blank comic thought balloons. It was explained that those children had heard the news about “the coronavirus”, and the students were asked to fill in their thoughts in the thought balloons. A total of 880 children completed the activity.

As shown in Table 4, COVID was the main concern (reported by 82.16% of the responders). This code comprised three subcodes: curiosity and concerns about the virus (“What is it?”; “Where does it come from?”), safety rules (“We will have to wear masks all the time”), and thoughts about a future cure and the development of the pandemic. The second notable code was connected to finding the positive side, hope, and reassurance (32.61%). It included items related to wishes of returning to a normal life (“I wish I could go back to school and see my friends”), rationalization about the opportunity to stay home with their own family (“At least I can stay home and play”), opportunities to sleep longer (“How lucky! I can sleep longer”), positive thoughts and wishful thinking (“We will make it through” or “Everything will be all right”). The third most prominent self-reported thoughts related to topics of death, disease, or pain (29.09%). These included concerns about the self (“I do not want to die” or “I do not want to catch COVID”), about family members (“What if my grandpa gets it”), and more general thoughts about others (“I hope none of my friends or relatives will catch it”). Other, less frequent thoughts were connected to the need to make sense of- and find meaning in the events (27.27%), with questions such as, “Why is this happening to me?”, or thoughts expressing their sense of loss for the things they were no longer able to do (25.91%), i.e., “I will not be able to go to the practice” or “I will miss meeting my friends at the park”, general fears (18.18%), anger (16.70%), thoughts about school distance learning (15.34%), and finally worries about things not returning to normal (13.30%).

**Table 4:** Students’ answers to the vignette, “John and Mary have heard of coronavirus from their parents and on television and have different thoughts. What are they thinking?” by school level and gender

Code	Full Sample % (*N* = 880)	Primary School		Middle School		Main effect of school		Main effect of gender		Interaction effect	
		Male % (*N* = 269)	Female % (*N* = 296)	Male % (*N* = 158)	Female % (*N* = 157)	χ^2^	*p*	χ^2^	*p*	χ^2^	*p*
COVID (overall)	82.16	81.04	85.14	77.85	82.80	1.04	308	2.91	0.088	0.00	0.951
*COVID: Curiosity and concerns*	65.91	65.80	65.54	64.56	68.15	nc	nc	nc	nc	nc	nc
*COVID: Safety rules*	41.14	37.92	46.62	38.61	38.85	nc	nc	nc	nc	nc	nc
*COVID: Cure and future of the pandemic*	5.34	5.95	3.72	6.96	5.73	nc	nc	nc	nc	nc	nc
Wishful thinking, finding the positive, hope,	32.61	26.39	24.32	37.34	54.14	38.19	**<** **0.001**	2.31	0.129	7.01	0.**008**
Death, disease, pain (of self, family or others)	29.09	28.25	24.66	35.44	32.48	5.47	**0.019**	1.21	0.271	0.03	0.863
Seeking sense and meaning of the event	27.27	21.19	25.34	28.48	40.13	12.41	**<** **0.001**	5.27	0.**022**	0.85	0.357
Feelings of loss, absence, loss of freedom	25.91	23.79	26.69	27.22	26.75	0.31	0.578	0.33	0.567	0.31	0.579
General fears	18.18	15.24	18.24	20.25	21.02	2.02	0.156	0.72	0.397	0.22	0.638
Anger, frustration	16.70	15.98	14.86	20.87	17.20	1.85	0.173	0.66	0.417	0.17	0.678
School, distance learning	15.34	9.29	11.82	24.68	22.93	26.11	**<** **0.001**	0.17	0.678	0.92	0.338
Worry about things not returning to normal	13.30	10.41	12.50	15.82	17.20	4.37	**0.037**	0.65	0.422	0.07	0.791
Other (including disbelief and aggressive thoughts)	8.98	7.06	4.73	20.89	8.28	nc	nc	nc	nc	nc	nc

Italicized text indicates sub-items; Bold text shows *p*-values < 0.05

While the main concern was the COVID pandemic (and it was equally reported across all school levels and genders), there were significant differences in the percentages of other codes between gender and school level. Indeed, thoughts related to finding the positive side, hope, reassurance, and positive expectations were reported more by MSS than PSS. In addition, a significant gender effect was observed between MSS and PSS students; female *vs*. male (54.14% *vs*. 37.34 %) in the MSS but female *vs*. male (24.32% *vs*. 26.39%) in the PSS. Furthermore, a higher percentage of MSS than PSS reported thoughts related to death, disease, pain, seeking sense and meaning, thoughts related to school distance learning, and worries about the difficulties of returning to normal life. Thoughts related to seeking sense and meaning were also reported by a higher percentage of females than males. Thoughts or feelings like sense of loss, lack of freedom, fear, and anger were equally reported across school level and gender.

RQ3 concerned children’s indications of activities they could do in class and with friends at the time of school re-entry (Table 5). RQ3 was based on a vignette representing a hot air balloon where children were invited to indicate their preferences. This activity aimed to encourage children to think of their everyday school life after the long isolation and to help teachers plan for the future. A total of 846 children completed the activity and were included in the following analyses. Most of the responders (86.64%) mentioned a school subject. The second most stated activity was connected to the concept of relationships and sharing things with friends (55.55%), and this included items such as “talking”, “going out”, “getting to know each other”; this activity was followed by play, joking, and laughing (51.42%), and break times (39.60%). While the two main preferred activities (school subject, and relationships and sharing things with friends) were equally reported across all school levels and genders, an effect was observed for school level in the two least reported activities (i.e., play, joking, laughing and break times). A higher percentage of PSS than MSS requested these activities (play *p* < 0.001, and break times *p* < 0.001).

**Table 5:** Students’ answers to the vignette, “Write on the balloon the things you will do together in school in the next days” by school level and gender

Code	Full Sample % (*N* = 846)	Primary School		Middle School		Main effect of school		Main effect of gender		Interaction effect	
		Male % (*N* = 261)	Female % (*N* = 291)	Male % (*N* = 150)	Female % (*N* = 144)	χ^2^	*p*	χ^2^	*p*	χ^2^	*p*
School subjects	86.64	85.44	89.69	84.00	85.42	1.34	0.247	1.95	0.163	0.47	0.495
Relationships, sharing with friends	53.55	50.57	54.30	52.00	59.03	0.74	0.391	2.02	0.156	0.22	0.640
Play, joking, laughing	51.42	58.62	59.45	40.00	34.03	37.56	**<0.001**	0.21	0.647	0.95	0.328
Recess time (Break time)	39.60	46.36	50.17	24.67	21.53	53.00	**<0.001**	0.18	0.670	1.03	0.311
COVID safety rules	7.80	6.13	6.53	14.00	6.94	nc	nc	nc	nc	nc	nc
Other (including negative feelings towards the school)	6.38	7.66	6.87	5.33	4.17	nc	nc	nc	nc	nc	nc

Bold text shows *p*-values < 0.05

## Discussion

The findings of this study indicate children’s concerns, how they coped with the lockdown, what they felt, and what they missed. The voices of these Italian school children raised both negative and positive aspects. Given that the content analysis includes both a qualitative and a quantitative phase, we will discuss our data in both ways.

From the qualitative point of view, our data revealed some rather novel information. This information came mostly from the class activity based on a “projective” vignette asking children to write the thoughts that two characters were having about the coronavirus during the lockdown. The central theme of the children’s reports was the need to make sense of the reality, by knowing and understanding it. A second theme was connected to positive thoughts and wishful thinking, and a third theme concerned fear of death and becoming ill. Naturally, the virus was the most recurrent thought that preoccupied the children because it was explicitly mentioned in the vignette. In our data, COVID was mentioned as part of a question about its nature and provenance and as part of a recap of the safety measures to prevent contagion.

Children’s determination to understand the virus is discernible as their need to know and make sense of the present. This can be perceived as a response to anxiety, were children try to compensate in a rational way for otherwise unknown aspects of their reality. In fact, sensemaking is a natural way to feel like we understand what is happening around us and as a function for keeping a certain level of control over ourselves and the environment (Proulx & Inzlicht, 2012). Indeed, feelings of uncertainty have been connected to the need to acquire and process information about the event that is causing it (van den Bos, 2009). As noted by Larsen et al. (2021), children’s concerns about the coronavirus seem to have direct connections with their emotional responses and worries for their families, and we also found that preoccupation with health and death was relatively high among our sample.

The next code on the projective vignette, in the order of relevance, was the one that grouped wishful thinking and other types of positive thoughts. Obviously, children need to counteract the danger and the unknown (represented by the crisis), and given that our vignette concerned mental reactions, after expressing their concerns, children turned to positive thoughts and wishful thinking. When the expectancy of meaning fails, people feel discomfort, and therefore initiate a set of emotional, cognitive, and behavioral compensatory actions to counteract such state of aversive affect that was prompted by the inconsistency between the individual and the reality (Larsen et al., 2021). Evidence suggests that disengagement strategies, including wishful thinking, may be preferable to engagement strategies in the context of perceived uncontrollable stressors such as the COVID-19 pandemic (Leitner et al., 2014). Additionally, the elaboration of positive thinking schemes strengthens children’s capacity to maintain a constructive perspective and deal effectively with stress, conflicts, or set-backs (Gilbert & Orlick, 2002). If wishful thinking is later associated with proactive coping activities such as engaging in social relationships or making themselves useful in the house, wishful thinking should be seen positively. An example of such activities in the school setting is provided by the original school program proposed by (Capurso et al., 2020).

Sense-seeking and sense-making thoughts and questions were not only connected to the virus but extended to more general contexts such as moment in time and daily life. These kinds of thoughts were more associated with MSS and females. This is in line with developmental literature showing that adolescents thinking becomes more abstract and hypothetical and that girls mature before boys (Marceau et al., 2011). To our knowledge, no other COVID-related studies have disclosed sense-seeking questions from children. These types of questions are usually overlooked in middle schools and crisis interventions. However, our findings provide a foundation to develop a curriculum and activities centered on the need to engage emerging adolescents’ abstract thoughts with more universal existential themes (Hacker, 1994).

The children clearly and consistently perceived the deep underlying danger of illness and death. As noted by Weaver and Wiener (2020), during the lockdown, children continually heard stories about illness and death that overrode the usual cultural tendency that would shield them from such a blunt reality. So, it is not surprising that these topics surface when their thoughts about the virus are elicited. While, in our sample, this code was higher for older children, anecdotal data show that this element was already present, even in children aged between two to four years (Pascal & Bertram, 2021). Not many COVID-related studies have shown fear of death in children. This is probably due to the tendency toward death denial in many Western societies, also common in Italy (Testoni et al., 2020). This content possibly emerged in our sample due to the projective nature of our prompt and the initial inductive phase of the content analysis procedure that allowed the researcher to identify this specific code even when death references were indirect.

On the quantitative side, our data appear to confirm other bodies of research in the field. The lockdown is connected to a deep experience of pervasive loss. Loss as a theme was consistently present in many different forms and across different types of the elicited narratives and codes. The children’s sense of loss encompassed physical, emotional, and existential aspects of their lives. It included their friendships, daily school life, freedoms, and family time with relatives. These items are discussed below.

Unsurprisingly, almost all the children missed their friends. Friendship plays a substantial role in children’s lives as a medium of support for coping and play (Waldrip et al., 2008). Our results suggest that the deep need for peer-based relationships was unfulfilled due to the social isolation measures and the absence of usual contact at school. The importance of the peer group becomes more internalized, starting at pre-adolescence when boys and girls start to separate from their families and engage more in a school-based social life. These results are in line with the findings of Larsen et al. (2021), who also identified that during lockdown children reporting missing their friends was associated with significantly higher self-reported emotional reactions. In line with contemporary literature on adolescence that sees friendship in a crucial developmental context (Crosnoe, 2000), friends were missed more by MSS. The fact that friends were missed so much during the first lockdown provides important information; namely, the technology-mediated relationships were perceived as insufficient surrogates for real-life friendships. When physically isolated from their friends, children missed – and indeed felt this loss – a number of social, emotional, and cognitive reciprocal developmental opportunities, in years crucial for individual’s development (see, e.g., Cavallina et al., 2018; Pedale et al., 2021). An example of this interpersonal dynamic lost during the lockdown can be observed in the code that reflected children missing hanging out and physical contact. Our findings show how identity and social relationships rely on a physical dimension made up of reciprocal contact, hugs, and routines, at least in Italian children and pre-adolescents.

School came next in the order of relevance, and children expressed missing it. The school code must be interpreted in its wider meaning, not solely in terms of academic education. In this context, school life represents the arena where students undertake sense-making of the events and actively take part in a social and developmental network (Reeves & Fernandez, 2016). At school, children form friendships, test their individuality with new tasks, and engage in relationships with adults other than the family (Larsen et al., 2021). When the lockdown was put in place, children and adolescents lost an important part of their life for a prolonged period. Missing school was reported significantly more by younger children and females, and these results confirm those of Kirsch et al. (2021) who reported a decreasing pattern of school satisfaction with age during COVID. One possible reason why girls miss school more than boys could be because they connected school life to emotional bonding and friendships. Additionally, girls also reported missing contact with family members and relatives and hanging out with their peers more frequently.

Freedom, autonomy, and independent leisure time are important achievements in adolescence and prepare young people for the transition to adult life (Soenens et al., 2017). This was another great loss for young people during the lockdown and was perceived significantly more by the MSS sample. No other studies have investigated this aspect of the lockdown which possibly emerged due to the open-ended nature of our method. Yet, losing their freedom was probably one of the main challenges faced by youngsters who responded to our survey. Just when they were starting to enjoy and explore more freedoms, 11- and 12-year-olds were drawn back to their rooms and forced to spend prolonged periods with their parents. Adolescence is a crucial time when youngsters explore their autonomy and independence (Leung & Shek, 2020). However, during the COVID pandemic their freedom and other unregulated, adult-unsupervised activities such as hobbies and sports, friendships and peers, hanging out and direct contact with others, were missed more by the older students.

Next to freedom, children also missed the opportunities to engage in sports and other forms of outdoor physical activities. This code showed a significant interaction between secondary school and male students, confirming the basic physical nature of many male leisure-time activities in adolescence (Al-Sobayel et al., 2015).

Despite the experience of loss being common and pervasive in their lives, children did mention a wide range of helpful resources. Here, the main overarching theme was relationships, but it did include one exception represented by the help perceived from video games and TV.

Family unanimously appeared as the main source of help across all school levels but with higher occurrence among girls in middle school. Our data confirm the great importance of family for the mental and physical wellbeing of children, and our findings are in line with the higher mean level of attachment of girls already identified by Buist et al. (2002). Other studies show that the lockdown also brought new and positive opportunities to children’s relationships with their parents and siblings (Mantovani et al., 2021). These studies confirm that children felt safe and protected in their home environment (Idoiaga et al., 2020). The children in our sample said that family members acted as a source of distraction and play and emotional and practical help. As positive as this information may be, it also raises the question of representativeness. In fact, our sample was limited to three school districts with all the children’s families living in a relatively privileged area (Umbria) compared to the average Italian family. Many families had grandparents living nearby. Moreover, given that the region is primarily countryside, many households had access to open spaces such as backyards or small vegetable gardens during the lockdown. Evidently, children who lived in less well-off areas of the country or families with compromised structures due to economic factors or intrafamily dynamics potentially missed out on this helpful resource (Save the Children Italia, 2020) and were not represented in this research.

Approximately 1/3 of the sample students reported video games and TV as sources of help, and this code was second only to family. Moreover, the use of video games and TV was prevalent in MSS and males. While extensive video game use and gender differences are not new concepts (Schleifer, 2018), the main question here is why and how TV and video games were perceived as help by our sample. The most immediate answer is that they probably filled the void left by the school and time with friends. The lockdown was long and boring, and boredom was a common phenomenon among lockdown children (Melegari et al., 2021). However, a deeper look into our data shows an ambivalent function of the use technology and related screentime. On one hand, technology may have been used as a means of positive communication. On the other hand, the intensive use of technology and video games did not fulfill the need of friendship and real-life social interactions because most children still reported missing their friends. Moreover, literature shows that excessive screentime can have harmful consequences such as sleep disruption, reduced motility, and increased risk of psychological complications (Lissak, 2018; Nagata et al., 2020), especially during the lockdown (Smirni et al., 2021). Fortunately, this passive role of the use of technology was not exclusive, and there were ample opportunities for other, more human, and empathic activities such as playing, helping with the house chores, or doing some physical activities. Information and communication technology (ICT) was not used passively. Our data also indicated an active use of ICT, with a marked prevalence in MSS and a slightly higher incidence in females. The active use of ICT code included its use for specific, developmental purposes; this was often seen in the need to connect and chat with friends or work on a hobby or project. Pre-adolescents develop higher mastery of the technological medium, and for them, this is often connected to their need for belonging, psychosocial wellbeing, and identity development (Allen et al., 2014).

The responses mentioning school and distance learning as a source of help were more expected and they supported the idea that children missed their school. As this code was evenly distributed among school levels and genders it suggests that, despite online limitations, the school’s presence was perceived by the all the students in our sample. School was often referred to as a source of relationships. Despite all the criticisms regarding the limitations of distance learning, the school guaranteed some continuity in education and development during the long days of the lockdown, and students felt its presence.

Play was seen as another source of help, especially among younger children, with no gender-related differences. Researchers have found that children play likely serves multiple interrelating adaptive and developmental functions, not only in building social, cognitive and problem solving skills, but also in helping with emotions and behavior regulation (Frankel, 2009, 2011; Hirsh-Pasek & Golinkoff, 2008; Russ, 1988). Capurso and Ragni (2016) similarly sustain that play serves an essential role in times of crisis and represents an important way of coping, particularly in younger children. In fact, according to the authors, children process reality through play; they find a safe ground where cognitive and affective processes are tested and faced. This allows children to moderate anxiety, aggression, frustration and conflict. Strayhorn (2002) and Russ (2004) claim that when children play, they are testing cognitive, behavioral, and emotional adaptive solutions within an imaginative frame. Such practice may help the child creating patterns of coping solutions that can be also expended in real life.

Our last activity asked children to express a wish connected to their return to school. Most children mentioned one or more school subjects, probably due to the school context and time (the beginning of a new school year) when the question was asked. The second recurring theme was friendships and social activities. These findings demonstrated the need to re-connect and re-build social relationships and confirmed that the school was perceived as a place where social relationships are fostered and realized (Eccles & Roeser, 2011). In younger children, this took the form of wanting free leisure time and play.

## Conclusion

This study listened directly to children’s voices and thoughts about the lockdown and addressed different associations based on gender and school level. It was a retrospective study carried out in the school setting once the first wave of the pandemic was over in Italy. The study used narrative excerpts taken from a set of post-lockdown school re-entry activities. Several concluding points can be outlined.

First, this research shows that children did experience this health crisis and its consequences first-hand, and at different levels – not only emotionally, but also physically and socially. Listening to and understanding the children’s voices helps us to identify strategies that could help them to deal with the crisis from psychological and social viewpoints (Idoiaga et al., 2020). Taken together, the data presented here show a complex array of positive and negative emotions, losses and coping strategies, relationships, and individual activities that were carried out during the lockdown. We believe that analysis of those things together, with a universal school population and in a daily school context, provided an overarching view of children’s thoughts and emotions.

Second, all the reported thoughts and emotions of worry, loss, and sense-seeking, reflect the children’s deep needs for open and inclusive communication and relationships with significant others in times of crisis. This task was often left to parents and families during the lockdown, while most schools concentrated on keeping up with the academic curriculum. However, it could also be undertaken with a proper school crisis management activity and as a part of the social-emotional curriculum.

Next, the children found many opportunities and activities to sustain their development and resilience during the lockdown. Some of them were able to find comfort at home from their family, or specific activities, even from their pets; others turned to school and distance learning, or friends, or discovered an active use of their technological devices. Our findings show how resourceful children can be, and that they can find strategies for coping and answers to their problems. The issue is that these resources were often found autonomously by the children and their families, which poses serious questions of equity of development opportunities.

The findings presented here deepen our knowledge of the dynamics connected to the effects of the COVID-19 crisis on children and young people. They also shed new light on tailoring crisis interventions programs based on age, developmental stage, and gender. The children’s narratives showed their strong will to continue with their lives, learn, stay in touch with their schools and peers, better understand what was going on, and play their part within their family and the community. These testimonies could provide a foundation for schools, educators, and administrators when planning crisis-related school-based interventions. They convey the key role that schools can have in maintaining, rebuilding, and planning for social and emotional connections between their community members, their environment, and their cultural background. They also exhibit how such activities should be delivered before or simultaneously with traditional subject-based curricular activities. Several examples of the activities can be found in the workbooks used in the present research (see the additional files section in Capurso et al., 2020). Regardless, each school should build its workbook based on the local culture, settings, and pedagogical organization. In sum, children’s accounts presented here manifest the value of their internal and social resources as well as their emotional and intellectual drive as active actors capable of instigating resilience in themselves and society in general. All these points should be taken into consideration by governments and local authorities to develop social and inclusive policies that address children’s psychological, social, and health needs to improve their ability to face future crises and the ever-changing world.

## Supplementary information

Supplementary File 1: Overview of the school-re-entry intervention for kindergarten children.

Supplementary File 2: Overview of the school-re-entry intervention for primary and middle school children.

Supplementary File 3: Codebook used for the content analysis with examples of the children’s answers

## Data Availability

The original Italian language datasets used and analyzed during the current study are available from the corresponding author on reasonable request.
