# Supplementary material for: Italian Children’s Accounts of the Lockdown: Insights and Perspectives
Source: J Child Fam Stud. 2023 Jan 10;32(1):145–59. doi: 10.1007/s10826-022-02508-6 (PMC9831020; doi:10.1007/s10826-022-02508-6)
Supplement: Supplementary file 1 — Overview of the school-re-entry intervention for kindergarten children. [file 10826_2022_2508_MOESM1_ESM.pdf]

Overview of the school-re-entry intervention for kindergarten children. Reprinted under original CC BY 4.0 license from (Capurso et al., 2020). The relating children's worksheet can be downloaded from https://doi.org/10.5334/cie.17.s3.

Objectives	Activity and Description	Rationale	Dimensions
Freely regain confidence with other schoolmates and with the school's spaces. Elicit pleasant moments relating to school attendance.	**1. Free play sessions, possibly outdoors**<br>Play has numerous benefits concerning stress (Fiorelli & Russ, 2012). Play allows children to find a natural form of expression, an arena where cognitive and affective processes are put in place. The first activity to do when children re-enter kindergarten is to let them play freely. This will allow them to regain confidence with the school spaces, classroom tools, and others.	Play is a key component of childhood development and can support coping capabilities (Capurso & Ragni, 2016; Gray, 2015)	Social, emotional, creative
Participate in a structured environmental exploration activity to regain confidence in the school's spaces. Foster children's awareness of themselves and others concerning their environment.	**2. Guided tour of school spaces**<br>Children are taken on a tour of the different areas in the school. For each room or school-designated space, children are asked to observe and list the different materials that characterize it. Teachers use probing questions and help the children re-organize the ideas and memories they already have. Teachers also help children to reconnect to that space with time references to remember what time during the day those specific spaces are used. Finally, teachers invite children to recall the aim of a particular school area and to share some fun event they were part of and that is connected to that space.	Young children develop a sense of self in time and space through environmental explorations (Hewes, 1982)	Spatial orientation; development of a sense of self in the context
Recognize that different emotions are a natural part of the self.<br>Realize that other children lived through the same type of events connected to the COVID-19 crisis.	**3. How did I feel at home?**<br>Teachers recap the fact that children had to stay at home with their parents because of the coronavirus emergency. They then show a set of feelings cards (see Appendix C) and ask pupils to:<br>– recognize the represented emotion;<br>– tell when, while they had to stay at home, they felt that specific emotion.<br>Given that there have been reports of increased incidents of domestic violence throughout the pandemic isolation, teachers should be aware of the fact that children may have witnessed or been a victim of violence. As with any other activity connected to family life, teachers should pay particular attention to signs of potential violence-related distress in children. In line with the local protocols and law, teachers should be ready to contact the local school and social authorities in case of signs or evidence that such violence occurred.	To process the emotions connected to the stressful events, children need to be able to share them with others and with adults within a setting that can provide a sense of protection (Theodore, 2016)	Emotion recognition and emotion processing

Evoke salient adults present at home with the family and share with classmates. Reinforce in children an awareness of the presence in their life of key adult figures that they can rely on.	**4. The important members of my family**<br>Trusted adults in the life of children are key figures for their emotional processing and represent a key factor that helps youngers to adjust to disruptions in their lives.<br>Teachers ask children to name family members that help them in their daily life or whenever they are facing difficulty. Teachers guide children with specific prompts to recall different situations (e.g., fear, daily life routines, homework, play and have fun).<br><br>After naming their significant family members, pupils are invited to:<br>1. Draw themselves and one of their family members as the adult is helping them with something.<br>2. Tell the class what they drew.<br>3. Tell the class what they were feeling when the family member was helping them.<br>4. Explain what the family member was doing and what he/she was feeling while helping the child out.<br>During the activity, the teacher outlines that, while the children's families can have a different configuration, each child can find, within his/her family, one or more adults that can help them when they need to face some problems.<br>To recap the activity, teachers can create a poster showing the different family figures (mothers, fathers, grandparents, siblings, uncles, etc.) and place next to them the child's name or a mark that identifies them.	Sharing and brainstorming with others allows children to reflect on different ways of coping with events and assess the consequences of different responses (Jimerson et al., 2005; Theodore, 2016)	Emotional and cognitive; family
Create a tangible reference to their own imagination.	**5. Draw a picture of the coronavirus.**<br>Children are asked to imagine the coronavirus and to draw it (see Appendix C).<br>This worksheet can also be used to identify what type of mental image the children have of the virus and give teachers perspective on how they understand it. Moreover, this worksheet prepares children for more cognitive work in Unit 7.	Various affective modulation and cognitive tools can be used to help children express their feelings and better understand the facts (Johnson & Figley, 1998; Theodore, 2016)	Concrete and creative

Make positive anticipatory thoughts and reconnect children with their school community.	**6. The story of the school of the block.**<br>The story of the school gives a common background and aims at allowing children to reconnect with the environment and each other. Additionally, the story elicits positive anticipatory thoughts in children towards the future. The story ends with opening up children's minds to their wishes and gives them a chance to express them and share with others.<br>After reading the story out loud, teachers can run a set of different activities connecting with it.<br><br>Activity 6.1 – Soap bubbles<br>Make sure to conduct this activity following the current safety measure as issued by your health care authority. The child doing the bubbles should be positioned far apart from the others; it would be preferable to do this activity outdoors.<br>Materials: Soap bubbles kit, one printed copy of the Story "At the School District" (see Appendix C).<br>Procedure: Read the story "At the School District" to the children, then explain that they can wish for something they want to do the next day and then blow it into the wind. Each child is called on to express his/her wish, tell it out loud, and then make a bubble to blow it in the air.<br><br>Activity 6.2 – Represent your wish<br>Children are invited to represent their wish with a small craft or a drawing. If the school has an outdoor area, it is advisable to let the children go outside to collect natural items for their craft. All the representations of the children's wishes are collected on a poster for display. On the following days, during circle time activities, teachers and children can choose different wishes to make them come true.<br>Children may receive a copy of the story to be taken home and to read within their family, to share and reinforce a sense of the future.	Use metaphor to process the current situation and direct attention to focus on positive thoughts and mental images (Theodore, 2016);	Emotional, creative, positive thoughts

Use practical strategies to prevent contagion.	**7. Wash your hands!**<br>Handwashing is one of the best ways to protect children from getting sick. Teaching when and how to wash hands is the most important activity to stay healthy.<br>Procedure: To introduce the activity, teachers remind the children about the Story "At the School District" (see Appendix C) and about the nasty little dot called coronavirus that jumped secretly from one person to the other. Then ask children if they know the meaning of the word "protect." Listen to children's responses and reformulate them to reflect the correct meaning. Then they ask if the children have any ideas on how to protect themselves from the coronavirus. Listen to the children's answers and underline the importance of handwashing. Then, they introduce the following activities:<br>Activity 7.1 – Let's get dirty!<br>Materials: Eco-friendly glitter, hand sanitizing gel or children's body oil, a sheet of paper, small plastic toys.<br>Procedure: Mix some lotion and glitter in a bowl, then have the children put some of the "germs" on their hands and rub them together. Invite them to touch some toys, the sheet of paper, and observe what happens to the "germs." Ask them what would happen if they touched their mouth or face now. Finally, ask them what to do to get rid of the "germs" from their hands and try out different suggestions until you get to the handwashing resolution. Have children wash their hands and point out what happens to the "germs."<br><br>Activity 7.2 – Make your handwashing poster<br>Procedure: Print one copy of Handout 7.2 for each child (see Appendix C). This is a blank page with a small logo that recalls the action of washing hands. Group children in twos and invite them to fill the page with a drawing or other picture of their own choice. For example, they could put in the contour or the stamp of their own hand or any other subject. When finished, laminate the sheets so they are water-resistant and post them in the bathrooms and other areas of the school to remind children to wash their hands often. If there are too many sheets, you can rotate them.<br><br>Activity 7.3 – Recap how to wash your hands<br>Using the poster provided (see Appendix C), recap with children the different steps of the handwashing procedure. You can then display the poster in the bathrooms and near other sinks to help children remember the correct method of handwashing.	Instructional programmes based on task demonstration and practical activities improve the quality of hand washing techniques.	Behavioral
To understand what germs are and use cognitive resources to understand the basic dynamics of microbiological life.	**8. Videos to know and understand germs**<br>In the weeks following school re-entry, it may be advisable to plan a science lesson about microorganisms. Children can learn about the pandemic by understanding microbial life. For younger students, two videos from Cincinnati Children's Hospital have been selected to help children understand what germs are and how they spread:<br>How Germs Spread	Explaining the Science for Kids<br>https://www.youtube.com/watch?v=YBGsoimPXZg<br>Stop Germs From Spreading:<br>https://youtu.be/JD85FDlxqCs<br>Once again, be careful not to exaggerate scary messages to prevent an increase in childhood anxiety.	Children can socially improve their understanding of the illness through interaction with peers and interactive school activities (Capurso et al., 2016)	Cognitive
