# Supplementary material for: Italian Children’s Accounts of the Lockdown: Insights and Perspectives
Source: J Child Fam Stud. 2023 Jan 10;32(1):145–59. doi: 10.1007/s10826-022-02508-6 (PMC9831020; doi:10.1007/s10826-022-02508-6)
Supplement: Supplementary file 2 — Overview of the school-re-entry intervention for primary and middle school children. [file 10826_2022_2508_MOESM2_ESM.pdf]

Table 1, *Overview of the school-re-entry intervention for primary and middle school children. Reprinted with permission (original CC BY 4.0 license) from (Capurso et al., 2020). The worksheet can be downloaded https://doi.org/10.5334/cie.17.s2.*

**Objectives**	**Activity and Description**	**Rationale**	**Dimensions**
Recall a salient moment in the time at home with the family and share it with the classmates.	**1. Draw a moment of these days**<br>With relation to the time spent at home due to the COVID-19 pandemic, children are asked to recall and draw a particular moment or event they experienced while confined at home (see Appendix B). The prompt is neutral (it does not say happy or sad moment), so that children are free to choose what they feel needs to be told. The teacher may use the content of the drawing (the type of scene, the emotions of the faces, and the moods of the characters) to get an initial sense of the children's feelings.	Children need to be able to rethink and share aspects connected to the stressful event to process their emotions (Theodore, 2016)	Social, familiar
Give opportunities for emotional processing of the past stressful situations and reflection on students' own and others' coping strategies.	**2. John and Mary's thoughts**<br>This worksheet operates at an emotional level. It is used to allow children to express thoughts and concerns they may have had during the coronavirus crisis when activities were interrupted and they had to live in isolation with their families.<br>Children are presented with a vignette that says that John and Mary have heard of the coronavirus from their parents and on television and have different thoughts (see Appendix B). Students are invited to write their thoughts on the vignette. This activity uses a "projective" methodology, as children are not asked directly what their feelings are, but instead, they project those feelings to the vignette's characters. The results can be shared with other schoolmates. During this activity, children may start to talk naturally about themselves. If this happens, they should be allowed to do it and let the conversation flow freely. Activity 3 must follow immediately after Activity 2.	Sharing and brainstorming with others allows children to reflect on different ways of coping with events and assess the consequences of different responses (Jimerson, Brock, & Pletcher, 2005; Theodore, 2016)	Emotional
	**3. When I'm worried**<br>After talking about the vignette's characters, children can now shift their attention to themselves. They are asked to list their common worries (not necessarily linked to the COVID-19 pandemic), but most important, they are redirected to think about their coping strategies and about the helping relationships they can rely on (see Appendix B). In terms of coping, these last two aspects are more important than fear or worrying. A sense of distress can be handled better when children perceive they have a set of resources that they can use to face the situation. So, when sharing the results of the activity, always point out the many strategies children will tell about. You may even set up a class poster to recap all the useful strategies and people children come up with.<br>This is a crucial exercise because it moves the focus of the child's mind from the problem to the coping strategy, so it is worth allowing enough time to it in the class.<br>Given that there have been reports of increased incidents of domestic violence throughout the pandemic isolation, teachers should be aware of the fact that children may have witnessed or been victim of violence. As with any other activity connected to family life, teachers should pay particular attention to recognizing signs of potential violence-related distress in children. In line with the local protocols and law, teachers should be ready to contact the local school and social authorities in case of signs or evidence that such violence occurred.		Cognitive-emotional
Create a tangible reference to their own imagination and use logical thought to recall useful	**4. Draw the coronavirus**<br>This activity works on an imaginative and cognitive level.<br>Children are asked to imagine the coronavirus and to draw it (see Appendix B). This worksheet can also be used to identify what type of mental image the children have of the virus and give teachers insight into how the students understand it. Moreover, the worksheet	Various affective modulation and cognitive tools can be used to help children express their feelings and better understand	Imaginative

Capurso, M., Pedale, T., Santangelo, V., Pagano Salmi, L., & Mazzeschi, C. (2023). Italian Children's Accounts of the Lockdown: Insights and Perspectives. *Journal of Child and Family Studies*. doi:10.1007/s10826-022-02508-6

strategies to prevent contagion.	prepares children to more cognitive work with Worksheet 5 and Unit 7 (see Appendix B).	the facts (Johnson & Figley, 1998; Theodore, 2016)	
	**5. What a forgetful guy!** Children are asked to recall three key recommendations to prevent contagion (see Appendix B). This worksheet is used to verify if and how children have internalized medical advice heard on TV. Teachers can use children's comments to reinforce positive behaviors and correct any incorrect statements. When commenting on healthy behaviors, teachers should always refer to updated governmental official recommendations. It is important to find a correct balance between recommended healthy behavior (e.g., hand washing, cleaning of personal devices) and the concept that microorganisms are a natural part of our environment and that many of them play a key role in sustaining human life along with the rest of the planet. Embracing correct behavior must not become an obsession with any microorganism.		Cognitive
Make positive anticipatory thoughts and reconnect children with their school community.	**6. Back to school again** This activity serves to create positive anticipatory thought and to reconnect the students with their schoolmates (see Appendix B). Now that they are back in school, children can go back to thinking about their relationships with others and all the activities they can do together in the following weeks.	Direct attention to focus on positive thoughts and mental images (Theodore, 2016)	Socio-relational
Know germs and use cognitive resources to understand the basic dynamics of microbiological life.	**7. Videos to know and understand germs** In the weeks following school re-entry, it may be advisable to plan a science lesson about microorganisms. Children can learn from the pandemic by studying and understanding microbial life.<br>For younger students, two videos from Cincinnati Children's Hospital Medical Centre have been selected to help them understand what germs are and how they spread;<br>How Germs Spread | Explaining the Science for Kids<br>https://www.youtube.com/watch?v=YBGsoimPXZg<br>Stop Germs from Spreading<br>https://youtu.be/JD85FDlxqCs<br>Once again, be careful to prevent increasing children's anxiety by avoiding exaggeration of scary messages.	Children can socially improve their illness understanding thorough peers and school interactive activities (Capurso, Lo Bianco, Cortis, & Rossetti, 2016)	Cognitive

Capurso, M., Pedale, T., Santangelo, V., Pagano Salmi, L., & Mazzeschi, C. (2023). Italian Children's Accounts of the Lockdown: Insights and Perspectives. *Journal of Child and Family Studies*. doi:10.1007/s10826-022-02508-6
