# Supplementary material for: Italian Children’s Accounts of the Lockdown: Insights and Perspectives
Source: J Child Fam Stud. 2023 Jan 10;32(1):145–59. doi: 10.1007/s10826-022-02508-6 (PMC9831020; doi:10.1007/s10826-022-02508-6)
Supplement: Supplementary file 3 — Codebook used for the content analysis with examples of the children’s answers [file 10826_2022_2508_MOESM3_ESM.pdf]

Codebook

Codes for the student answers to the question "What did you miss most during the lockdown?".

Code	Description	Inclusion and exclusion criteria	Examples
Friendship - Peers	Explicit references to schoolmates, friends, peers or to the concept of friendship.	Inclusions (I.) people names, schoolmates, friends, references to the class as a social context.<br>Exclusions (E.)<br>Siblings, pet names, physical contact with peers	*meeting with my friends.*<br>*my friend Sofia.*<br>*my classroom friends.*
School	References to school activities or school personnel	I. school, classroom and teachers references; doing a school activity,<br>E. schoolmates.	My teachers and my class.<br>*Going to school.*<br>*Math and Italian.*
Freedom, autonomy	Direct or indirect indications connected to freedom or autonomy.	I. Outdoor and autonomous activities that were done without adult supervision.<br><br>E.: hobby, sport.	*Going out.*<br>*Go to play in the park*<br>*Freedom*<br>*Could not go out.*
Family, relatives	Explicit references to relationships with members of the extended family	I. relatives such as Mothers and fathers, grandparents, uncles, cousins etc.	Visit my grandma.<br>my cousins.<br>freetime with my dad.
Sports – Hobbies - Physical activity	References to all formal sports and other informal leisure physical and outdoor activities.	I. sports, riding the bike, outdoor activities.<br>E. generic hanging out references.	I missed volleyball.<br>going out for a walk.<br>going to Salsa practice soccer.<br>going to the swimming pool<br>Wandering around with my bike
Hugs/hugging - physical contact-<br><br>Gathering/ hanging out with friends	References to contexts that favor meeting other people or allow physical contact.	I. meeting, hugging, touching, kissing, etc.<br>E. generic references to friends / friendships.	touching my friends<br>hugging my grandparents<br>walking holding hands with my best friend.<br>kissing hello.

Capurso, M., Pedale, T., Santangelo, V., Pagano Salmi, L., & Mazzeschi, C. (2023). Italian Children's Accounts of the Lockdown: Insights and Perspectives. *Journal of Child and Family Studies*. doi:10.1007/s10826-022-02508-6

Play	Explicit references to the lack of recreational activities in all their forms	I. group games, outdoor games, the concept of having fun, etc.	Play with my friends.<br>Play outside.<br>Have fun<br>Play together
Other (including Pets and positive feelings)	Other		
	Positive feelings	I. references to positive feelings or positive interpersonal atmosphere.	happiness<br>our harmony<br>smiles on my friends faces
	Pets	I. All species of pets or mentioning pet names.<br>E.	My grandma dog<br>My horse<br>Alice's cat

Capurso, M., Pedale, T., Santangelo, V., Pagano Salmi, L., & Mazzeschi, C. (2023). Italian Children's Accounts of the Lockdown: Insights and Perspectives. *Journal of Child and Family Studies*. doi:10.1007/s10826-022-02508-6

Codes for the student answers to the question "What helped you most during the lockdown?"

Code	Description	Inclusion and exclusion criteria	Examples
(Support from)<br>Family members and other relatives	References all figures within the family context who supported the children during the early stages of the Covid emergency.	Inclusions (I.): the presence of or activities carried out with members of their family unit<br>Exclusions (E.)Proper personal names.	My parents and my sister<br>Being with my parents<br>Grandparents<br>Mom and dad
Videogame, TV (passive)	Explicit references to the passive use of technology to play or watch videos as entertainment	I. Videogames, gaming platforms, apps and services that involve watching videos<br>E. Includes: video games, gaming platforms, service names or applications that involve watching videos<br>Excludes: Using devices to have meaningful relationships	Netflix<br>My Playstation<br>NIntendo Switch<br>Watch so much TV
Distance learning	Explicit references regarding (operators) in the school context, to all activities and subjects carried out during distance learning.	I.: teachers, assistants, school subjects, school activities, diverse ways of connecting online?<br>E.	Video Lessons<br>Distance lessons<br>Homework<br>The teacher
Active use of information and communication technology (ICT)	References to the active use of technology to keep in touch with other people, to carry out research or to produce videos for entertainment.	I. video editing activities, making calls or sharing information through virtual meeting platforms, using search engines to get information<br>E. video lessons, videogames or watching television	Video calls with friends<br>Phone<br>Computer<br>Internet
Play	Explicit references to recreational or play activities carried out in all their forms to pass time or build relationships	I. Recreational or play activities carried out alone, with other people or animals, whether they are outdoors or indoors.<br>E. games conducted via digital platforms or when the child uses the word game to indicate a sport.	Play with my younger brother<br>Lego<br>Played with toys<br>Play with dolls

Capurso, M., Pedale, T., Santangelo, V., Pagano Salmi, L., & Mazzeschi, C. (2023). Italian Children's Accounts of the Lockdown: Insights and Perspectives. *Journal of Child and Family Studies*. doi:10.1007/s10826-022-02508-6

Pets	References to animals that have helped children to "tolerate" everyday life during the Covid emergency.	I. animal names, playtime with animals, pets, animals belonging to relatives or friends.	Cats<br>Playing with my pets<br>My dog "Gea"<br>Grandma's dog
Friendship	Explicit references to classmates or peers with whom a friendship has been established.	I.: friends, personal names, relationships also through digital channels, etc.<br>E.	My friends<br>Talking with friends<br>Friends seen virtually<br>Classmates-peers
Sports – Hobbies - Physical activity	Explicit references to all activities carried out individually or in groups, outdoors or indoors, which produce a psycho-physical well-being in which commitment and passion are invested/involved.	I., playing sports, swimming pool, walking, gym, bike, playing soccer, drawing, ballet, etc.<br>E.	Playing soccer in the garden<br>riding bikes<br>Going for a walk<br>Drawing
Feeling useful around the house	References all those situations in which the child felt useful in carrying out household activities alone or with a family member	I. household activities done alone or with a parent<br>E.	Helping mom around the house<br>Make lunch<br>Prepare desserts<br>Helping my dad with the lawn
Places and resources within the house	Explicit references to all areas of the house used as a mean to cope with the Covid emergency	I. rooms in the house, garden outside the house, your own swimming pool, furniture such as a bed or sofa<br>E. swimming pools intended as places of leisure or sports outside the home environment	The Sofa<br>I went to the balcony<br>The home pool<br>Garden
Reading,	Explicit references to spending time engaged in reading books for fun	I. reading books, reading comics, etc.<br>E. reading school books	Read a book<br>Reading helped me<br>Manga<br>The books
music	Explicit references to individual pleasure in listening to music or playing a musical instrument	I. listening to music, practicing with an instrument.	Listening to music<br>The flute<br>The song that Lisa dedicated to me<br>Music

Inner strength and self resources	Explicit references to the individual's internal psychological resources that help him cope with life situations.	I. being confident, have courage, patience, etc.<br>E.	Hope that things improve<br>Be brave<br>patience
Other	Indicates all those words and phrases that were difficult to interpret or that did not fit into a specific Code	I.: non-decipherable terms, random words, responses inconsistent with the request, or words that indicate emotional states.<br>E. all terms that fell within a specific Code	None<br>Scared<br>The presents they made for me<br>Being alone

Codes for the student answer to the vignette "Write on the balloon the things you will do together in school in the next days", by school level and gender (Table 5)

	Code	Description	Inclusion and exclusion criteria	Examples
	School subjects	References to all those activities and disciplines carried out and learned by children within the school context	Inclusions (I.)peer-to-peer activities, teaching subjects, school trips, learning activities.	Do homework together<br>Studying new things<br>Do gymnastics class<br>Math
	Relationships, sharing with friends	References to those actions that promote relationships with classmates or teachers	I. every time the word "together" was written it was considered a relationship, collective activity	Meet new people<br>Talk together<br>Have fun all together<br>Talk about our personal thoughts
	Play, joking, laughing	Explicit references to playful-recreational activities carried out in all their forms to pass the time or promote relationship building in the school context.	I. fun activities, jokes, games of various kinds.	Laugh<br>To play<br>Joke<br>To play hide and seek
	Recess time	References to all those moments carried out within the school walls, but which go beyond teaching	I. recess time, going outdoors,	Recess time<br>We went outside<br>Eat together<br>We celebrate birthdays
	Covid safety rules	References to all those actions used to safeguard and protect one's own health and of others within the school context	I. activities carried out to talk about Covid	We just have to keep our distance<br>Disinfect your hands<br>We will remind each other of the rules<br>Play one meter apart from each other
	Other	Indicates all words and phrases that were difficult to interpret or that did not fit into a specific Code	I. terms that cannot be deciphered, random words, answers that are not consistent with the request<br>E. all terms that fell within a specific Code	Watch the hummingbird butterflies flying<br>We will be four<br>To smile<br>I don't know

Capurso, M., Pedale, T., Santangelo, V., Pagano Salmi, L., & Mazzeschi, C. (2023). Italian Children's Accounts of the Lockdown: Insights and Perspectives. *Journal of Child and Family Studies*. doi:10.1007/s10826-022-02508-6

[illegible]	Negative feelings	References to all words used by children to describe back to school in a negative sense	I. boredom, anger manifestations<br>E. positive feelings	We will get angry<br>Get bored<br>Argue<br>We will get bored

Capurso, M., Pedale, T., Santangelo, V., Pagano Salmi, L., & Mazzeschi, C. (2023). Italian Children's Accounts of the Lockdown: Insights and Perspectives. *Journal of Child and Family Studies*. doi:10.1007/s10826-022-02508-6

Codes for the Student answer to the vignette *"John and Mary have heard of Coronavirus from their parents and on television and have different thoughts. What are they thinking?"*.

	Code	Description	Inclusion and exclusion criteria	Examples
	Covid: safety rules	Explicit references to protective behaviors normally indicated to prevent Covid infections	Inclusions (I.): social distancing, hygiene rules, masks, etc.<br>Exclusions (E.) vaccine, medical therapies, etc.	you have to wear a mask!<br>We can't hug each other!
	Covid: cure and future of the pandemic	References to future or upcoming cures and therapies to defeat Covid infections	I. vaccine, remedies, medical treatment, etc.<br>E. symptoms, disease, pain	When will they find the vaccine?<br>Is there a cure?<br>Will they find a remedy?<br>Doctors must make a move to find the covid-19 vaccine
	Covid: curiosities and concerns	Description and questions related to the nature of the virus and the danger of covid.<br>The main subject is the virus / pandemic.	I. nature, general curiosities, evolution of the virus<br>E. care, general requests for meaning	What is it?<br>How did it develop?<br>It's too contagious<br>Why is it called covid-19?
	Hope, reassurance<br>Finding the positive	Questions related to the future, desire for normality and return to the life before the covid	I. , questions about what will happen next or in the future<br>E. missing out, worries of not returning to normality, intolerance	What will happen next?<br>Will I go back to school to study and play with my friends<br>When will we be able to leave the house?
		Confident state of mind, hopeful expectations and wishes that the situation can improve	I.: optimism, wishful thinking<br>E. disbelief, worry no return to normal.	But I'm sure we'll make it!<br>It will all be fine<br>I hope it ends soon and that we can live normally
		Indicates statements relating to the positive aspects of the present situation	I. positive reframing of the present<br>E. hope, future positive expectations	Let's have fun!<br>At least I can play video games<br>At least I can sleep more<br>Lucky for netflix

Capurso, M., Pedale, T., Santangelo, V., Pagano Salmi, L., & Mazzeschi, C. (2023). Italian Children's Accounts of the Lockdown: Insights and Perspectives. *Journal of Child and Family Studies*. doi:10.1007/s10826-022-02508-6

	Death, disease, pain of people outside the immediate family	Worry about death, pain or illness in reference to others	I. Other people getting injured, sick or dying.<br>E. loss, fear, worry, self and family death	How many people have died from the coronavirus? How many were hospitalized instead?<br>There are some who heal others who die<br>People are sick
	Death, disease, pain of family members	Worry about death, pain or illness in relation to family members	I. Family members getting sick, injured, in pain, or dying.<br>E. fear, loss, worry	What if the coronavirus infects our family?<br>But I wonder if it could affect my family and it could take away my parents<br>What if anyone in our family dies from the coronavirus?
	Death, disease, pain of self	Worry about death pain or illness related to oneself	I. injuries and pains of the self, getting sick or dying.<br>E. general fear, worry, loss	I don't want to die<br>What if I have the virus?<br>I don't want to get sick with coronavirus!
	Seeking sense and meaning	Wondering why and what is happening in order to understand the meaning of current events.	I. general questions<br>E. protective measures, questions about the nature of the virus	Why is everyone talking about this?<br>What is happening?<br>Why do we have to stay at home?
	Bans/loss/absence	References to the restriction to not carry out certain activities based on what is established by an authority, claims relating to loss and shortcomings.	I. limitations of personal life, a sense of emptiness, loss of everyday life, lack, nostalgia<br>E. nature, no return to normality, reference to the future	Because this coronavirus we had to stay at home<br>I can't go to the park<br>I miss my friends
	General fears	Indicates the emotional state of apprehension in relation to a presumed or real danger	I. worry, fear, anxiety<br>E. concern for the future, for covid, illness and pain	I am really worried<br>I am very worried! It makes me so afraid and worried because it could affect me too<br>anxiety

Capurso, M., Pedale, T., Santangelo, V., Pagano Salmi, L., & Mazzeschi, C. (2023). Italian Children's Accounts of the Lockdown: Insights and Perspectives. *Journal of Child and Family Studies*. doi:10.1007/s10826-022-02508-6

	Anger / frustration	Feeling of inner discomfort connected to the current condition of discomfort	I. boredom, annoyance, intolerance<br>E. aggression, disbelief, fear.	I won't be able to see anyone anymore, I think I'll go crazy<br>I will be very bored<br>Always go out with a mask<br>It seems like a nightmare coming true
	Distance learning (how will it work)	Doubts and perplexities concerning the school and the telematic modality	I. school area<br>E. request for meaning, questions relating to the nature of the covid	How do I do with the school?<br>What does remote school mean?<br>Will the benches be far apart?
	Worry about things not returning to normality	Questions about the future with the doubt that certain things will never return to "normal"	I. sentences with adverbs never and forever, reference to the future,<br>E. shortcomings, loss of everyday life	Will we ever return to indoor sports?<br>Will the covid be there forever?<br>Should we stay home all our life?
	Other/non specified	Statements that did not fit a specific code		Good morning from Mondello, today we are at the beach, there is no "coviddi"<br>Never seen ads before<br>Or about a new football team

Capurso, M., Pedale, T., Santangelo, V., Pagano Salmi, L., & Mazzeschi, C. (2023). Italian Children's Accounts of the Lockdown: Insights and Perspectives. *Journal of Child and Family Studies*. doi:10.1007/s10826-022-02508-6
